# The impact of complex informative missingness on the validity of the transmission/disequilibrium test (TDT)

**DOI:** 10.1186/1753-6561-1-s1-s26

**Published:** 2007-12-18

**Authors:** Chao-Yu Guo

**Affiliations:** 1 Department of Mathematics and Statistics, Boston University, Boston, Massachusetts and National Heart, Lung and Blood Institute, Framingham Heart Study, Framingham, Massachusetts 01702, USA

## Abstract

The transmission/disequilibrium test was introduced to test for linkage and association between a marker and a putative disease locus using case-parent triads. Several extensions have been proposed to accommodate incomplete triads. Some strategies assumed that parental genotypes were missing completely at random and some methods allowed informative missingness for parental genotypes. However, the above tests assumed that offspring genotypes were missing completely at random and concluded that the transmission/disequilibrium test remained a valid test by excluding incomplete triads from the analysis. In this article, the conditional distribution of ascertained triads allowing informative missingness for offspring genotypes, as well as their parental genotypes, was derived and several tests under such scenarios were evaluated. In simulations, independent triads from the Genetic Analysis Workshop 15 simulated data (Problem 3) was ascertained. When offspring genotypes were missing informatively, simulation results revealed inflated type I error and/or reduced power for the transmission/disequilibrium test excluding incomplete triads.

## Background

Recently, family-based association studies have drawn substantial attention in genetic studies as a way to avoid spurious association due to population admixture. The transmission/disequilibrium test (TDT) by Spielman et al. [1] was proposed to test for linkage and association between a marker and a disease locus using ascertained case-parent triads. However, parental genotypes may be unavailable due to refusals or other unknown causes. Assuming that only one parental genotype is available and the other one is missing completely at random (MCAR), Clayton [2] and Weinberg [3] proposed likelihood ratio tests and Sun et al. [4] introduced the TDT with only one parent is available (1-TDT) to incorporate such dyads (affected offspring with one parental genotype). Later, the expectation maximization algorithm based haplotype relative risk (EM-HRR) proposed by Guo et al. [5] extended the haplotype relative risk (HRR) test [6] to accommodate both dyads and monads (affected offspring without parental genotype). However, when missingness cannot be ignored (i.e., a missing pattern of parental genotypes is related to the disease under study), the assumption of MCAR is violated and these tests may be invalid.

When parental genotypes are missing informatively, Allen et al. [7] and Chen [8] proposed likelihood ratio tests to assure the validity of testing for association between a candidate gene and a disease. However, the cost of accounting for informative missingness is reduced power. When the missing pattern was indeed completely at random, one can see that Allen et al.'s strategy could be less powerful than the 1-TDT [7]. This is also true for Chen's method (see Table 4 [8]). The power of Chen's score statistic with 1 degree of freedom is less than that of the TDT using only intact triads for a common (rare) allele under the dominant (recessive) disease model, as is the score statistic with 2 degrees of freedom for both rare and common variant alleles under the multiplicative inheritance. This means that the inclusion of dyads reduces the power of the score test in these cases.

Regardless of different missing patterns among parental genotypes, the above-mentioned methods assumed that offspring genotypes were MCAR. In the following, the conditional distribution of ascertained triads that allows informative missingness for offspring genotypes will be derived, as well as their parental genotypes, and several tests under such scenarios will be evaluated.

## Methods

### Distribution of ascertained triads

First, it was assumed that the data consisted of genotypes of bi-allelic markers such as a single-nucleotide polymorphism (SNP). Therefore, there are exactly two alleles, B_1 _and B_2_, at the marker locus. The distribution of complete triads was derived as the following: Let G_o_, G_pf_, G_pm _be the offspring's, father's, and mother's genotypes, respectively. Let G_of _and G_om _be the offspring allele inherited from the father and mother, respectively. Here, imprinting was not considered, and the four possible joint probabilities of a given parental genotype and the probability of transmitting a given allele to the offspring from that parent, all conditional on offspring affected status are:

*μ *= Pr{[G_f _= (B_1_B_1_) & G_of _= (B_1_)] or [G_m _= (B_1_B_1_) & G_om _= (B_1_)]|affected offspring}

*υ *= Pr{[G_f _= (B_1_B_2_) & G_of _= (B_1_)] or [G_m _= (B_1_B_2_) & G_om _= (B_1_)]|affected offspring}

*ζ *= Pr{[G_f _= (B_1_B_2_) & G_of _= (B_2_)] or [G_m _= (B_1_B_2_) & G_om _= (B_2_)]|affected offspring}

*τ *= Pr{[G_f _= (B_2_B_2_) & G_of _= (B_2_)] or [G_m _= (B_2_B_2_) & G_om _= (B_2_)]|affected offspring}.

When the disease model is recessive, Ott (Table 2, [9]) showed that *μ *= (*s *+ *δ*/*r*)*s*, *ν *= (*s *+ *δ*/*r*)(1 - *s*) - *θδ*/*r*, *ξ *= (1 - *s *- *δ*/*r*)*s *+ *θδ*/*r *and *τ *= (1 - *s *- *δ*/*r*)(1 - *s*), where *r *is the allele frequency of the recessive disease allele, and *s *is the allele frequency of marker allele "B_1_". The parameter *θ *denotes the recombination fraction, and *δ *= *p*(*aB*_1_) - *p*(*a*)*p*(*B*_1_) denotes the disequilibrium coefficient between the marker and the disease locus.

Let I_f_, I_m _and I_o _be binary indicator functions for father, mother, and offspring having missing genotype information. For example, I_f _= 1 if the father's genotype is missing and 0 otherwise. Let P_o11_, P_o12_, and P_o22 _denote missing rates for offspring with B_1_B_1_, B_1_B_2_, and B_2_B_2 _genotypes, respectively. Similarly, let P_f11_, P_f12_, and P_f22 _(P_m11_, P_m12_, and P_m22_) denote missing rates for father (mother) with B_1_B_1_, B_1_B_2_, and B_2_B_2 _genotypes, respectively. Note that we do not assume any pattern for the nine missing parameters, i.e., missingness of a given parental genotype can be dependent or independent of the other parent's and/or offspring's genotype. Assuming random mating, one can calculate the conditional probability of ascertaining a complete triad with the father, mother, and affected offspring's genotypes being B_1_B_1_, B_1_B_2_, and B_1_B_2_, respectively, as

Pr(L_f _= 0 & G_f _= (B_1_B_1_); I_m _= 0 & G_m _= (B_1_B_2_); I_o _= 0 & G_o _= (B_1_B_2_)|affected offspring) = *μ *× *ζ *× (1 - P_f11_) × (1 - P_f12_) × (1 - P_o12_).

The distribution of remaining ascertained triads can be derived in a similar manner and is displayed in Table 1. P_*k*_^i, j ^and M_*k*_^i, j ^are the conditional probability and observed counts for each type of triad data, where *k *= "0", "1", or "2" represents the total number of B_1 _alleles transmitted to the offspring, and *i*, *j *= "0", "1", or "2" represents the total number of B_1 _alleles for fathers and mothers, respectively.

**Table 1:** Distribution of ascertained triads

Affected offspring	Father	Mother	Probability	Obs.
B_1_B_1_	B_1_B_1_	B_1_B_1_	P_2_^2,2 ^= *μ*^2 ^× (1-P_f11_) × (1-P_m11_) × (1-P_o11_)	M_2_^2,2^
	B_1_B_1_	B_1_B_2_	P_2_^2,1 ^= *μν *× (1-P_f11_) × (1-P_m12_) × (1-P_o11_)	M_2_^2,1^
	B_1_B_2_	B_1_B_1_	P_2_^1,2 ^= *μν *× (1-P_f12_) × (1-P_m11_) × (1-P_o11_)	M_2_^1,2^
	B_1_B_2_	B_1_B_2_	P_2_^1,1 ^= *ν*^2 ^× (1-P_f12_) × (1-P_m12_) × (1-P_o11_)	M_2_^1,1^
				
B_1_B_2_	B_1_B_1_	B_1_B_2_	P_1_^2,1 ^= *μζ *× (1-P_f11_) × (1-P_m12_) × (1-P_o12_)	M_1_^2,1^
	B_1_B_2_	B_1_B_1_	P_1_^1,2 ^= *μζ *× (1-P_f12_) × (1-P_m11_) × (1-P_o12_)	M_1_^1,2^
	B_1_B_1_	B_2_B_2_	P_1_^2,0 ^= *μτ *× (1-P_f11_) × (1-P_m22_) × (1-P_o12_)	M_1_^2,0^
	B_2_B_2_	B_1_B_1_	P_1_^0,2 ^= *μτ *× (1-P_f22_) × (1-P_m11_) × (1-P_o12_)	M_1_^0,2^
	B_1_B_2_	B_1_B_2_	P_1_^1,1 ^= 2*νζ *× (1-P_f12_) × (1-P_m12_) × (1-P_o12_)	M_1_^1,1^
	B_1_B_2_	B_2_B_2_	P_1_^1,0 ^= *ντ *× (1-P_f12_) × (1-P_m22_) × (1-P_o12_)	M_1_^1,0^
	B_2_B_2_	B_1_B_2_	P_1_^0,1 ^= *ντ *× (1-P_f22_) × (1-P_m12_)× (1-P_o12_)	M_1_^0,1^
				
B_2_B_2_	B_1_B_2_	B_1_B_2_	P_0_^1,1 ^= *ζ*^2 ^× (1-P_f12_) × (1-P_m12_) × (1-P_o22_)	M_0_^1,1^
	B_1_B_2_	B_2_B_2_	P_0_^1,0 ^= *ζτ *× (1-P_f12_) × (1-P_m22_) × (1-P_o22_)	M_0_^1,0^
	B_2_B_2_	B_1_B_2_	P_0_^0,1 ^= *ζτ *× (1-P_f22_) × (1-P_m12_) × (1-P_o22_)	M_0_^0,1^
	B_2_B_2_	B_2_B_2_	P_0_^0,0 ^= *τ*^2 ^× (1-P_f22_) × (1-P_m22_) × (1-P_o22_)	M_0_^0,0^

Total			$Sum=\sum_{i,j,k}P_{i}^{j,k}$	N_triads_

### Validity of the TDT under various missing patterns

As shown in Table 1, the conditional probability of a heterozygous parent transmitting the B_1 _(B_2_) allele to the affected offspring was calculated as $T_{1}=\frac{P_{2}^{2,1}}{2}+\frac{P_{2}^{1,2}}{2}+P_{2}^{1,1}+\frac{P_{1}^{1,1}}{2}+\frac{P_{1}^{1,0}}{2}+\frac{P_{1}^{0,1}}{2}(T_{2}=\frac{P_{0}^{1,0}}{2}+\frac{P_{2}^{0,1}}{2}+P_{0}^{1,1}+\frac{P_{1}^{1,1}}{2}+\frac{P_{1}^{2,1}}{2}+\frac{P_{1}^{1,2}}{2})$. When there is no linkage or no association, *T*_1 _= *T*_2_, if and only if offspring genotypes are missing completely at random (*P*_*o*11 _= *P*_*o*12 _= *P*_*o*22_). Therefore, when offspring genotypes are missing informatively (at least two of *P*_*o*11_, *P*_*o*12_, and *P*_*o*22 _are not equal), the TDT does not provide a valid test for linkage and association by excluding incomplete triads from the analysis (*T*_1 _≠ T_2_). Such phenomenon is also true for the HRR proposed by Falk and Rubinstein [6], which is a valid test for association in the presence of linkage.

### Simulations

Unrelated nuclear families were used each with two affected siblings and complete parental genotypes from the Genetic Analysis Workshop 15 simulated data (Problem 3). Based on the 100 replicates provided, the first 10 replicates were pooled together. To assure the assumption of independence among ascertained triads, we randomly selected only one affected offspring from each nuclear family to form the new population for simulations. In order to reflect realistically complex disease models, missing status for the affected offspring and their parents was assigned. The missing patterns considered were the recessive, dominant, and additive genetic effect models for both major and minor alleles as indicated in the second column of Table 2. Therefore, only a proportion of families with an affected offspring were eligible for the ascertainment and the total number of families ascertained including triads, dyads and monads were 200.

"SNP6_150" on chromosome 6 and "SNP15_55" on chromosome 15 were used in power and type I error simulations, respectively. Several other SNPs were also considered but with similar results and the results are not shown here. For SNP6_150 (SNP15_55), genotype frequencies are 0.41 (0.31) for major homozygote, 0.46 (0.50) for heterozygote, and 0.13 (0.19) for minor homozygote. A total of 1000 repetitions were conducted for power and type I error simulations. The TDT and HRR were applied to the subset of complete triads. The 1-TDT [4] and EM-HRR [5] were both applied to the subset of complete triads and dyads.

**Table 2:** Simulation results

	Missing patterns	Chromosome 15 (type I error: SNP15_55)				Chromosome 6 (power: SNP6_150)			
Model	(P_f11_, P_f12_, P_f22_)(P_m11_, P_m12_, P_m22_)(P_o11_, P_o12_, P_o22_)	TDT	1-TDT	HRR	EM-HRR	TDT	1-TDT	HRR	EM-HRR
1	(0.2,0.2,0.2) (0.2,0.2,0.2) (0.2,0.2,0.2)	5.3	4.8	5.2	4.5	21.5	25.7	22.6	27.9
									
2	(0.4,0.2,0.2) (0.4,0.2,0.2) (0.1,0.1,0.1)	4.7	16.5	5.8	19.7	21	67.1	22.3	73.9
2	(0.4,0.4,0.2) (0.4,0.4,0.2) (0.1,0.1,0.1)	5.8	14.3	4.4	12	13.8	43.7	11.9	42
2	(0.4,0.3,0.2) (0.4,0.3,0.2) (0.1,0.1,0.1)	5.1	14.4	4.5	16.2	15.9	58	15.7	60.7
2	(0.2,0.2,0.4) (0.2,0.2,0.4) (0.1,0.1,0.1)	4.3	5.1	4.6	6.2	20.1	20.9	21.5	20.7
2	(0.2,0.4,0.4) (0.2,0.4,0.4) (0.1,0.1,0.1)	4.5	6.4	4.1	8.9	15.4	7.7	12.9	5
2	(0.2,0.3,0.4) (0.2,0.3,0.4) (0.1,0.1,0.1)	5.1	6.9	5.3	8.6	17.3	12.6	16.4	10.9
									
3	(0.4,0.2,0.2) (0.4,0.2,0.2) (0.4,0.2,0.2)	9.5	3.6	10.8	5.5	4.9	21.7	5.5	27.4
3	(0.4,0.4,0.2) (0.4,0.4,0.2) (0.4,0.4,0.2)	9.6	4.2	8.5	4.7	7.9	20.2	7	19.7
3	(0.4,0.3,0.2) (0.4,0.3,0.2) (0.4,0.3,0.2)	8.6	3.4	8.6	4.4	6	18.6	6.1	22.1
3	(0.2,0.2,0.4) (0.2,0.2,0.4) (0.2,0.2,0.4)	10	5.4	11.9	7	34.1	24.8	36.8	29.9
3	(0.2,0.4,0.4) (0.2,0.4,0.4) (0.2,0.4,0.4)	13.5	4.7	12.2	5	41.7	22.2	39.5	23.1
3	(0.2,0.3,0.4) (0.2,0.3,0.4) (0.2,0.3,0.4)	11.5	3.8	11.6	3.6	40.1	23.5	39.7	26.4

## Results

In Table 2, the first column indicates the model of missingness (1, MCAR for all genotypes; 2, informative missingness for parental genotypes and MCAR for offspring genotypes; 3, informative missingness for all genotypes). The three brackets in the second column display missing rates for the father, mother and offspring, respectively. The results in the first seven rows indicate that, when offspring genotypes are MCAR, the TDT and HRR are valid tests at 5% nominal level as seen in Guo et al. [10]. However, the 1-TDT and EM-HRR were invalid due to inflated type I error over the nominal level when parental genotypes are missing informatively (row 2–7), which matches the results in Allen et al. [7] and Chen [8]. In addition to previous findings, we also discovered that power of the 1-TDT and EM-HRR can be not only inflated (row 2–4), but also reduced (row 5–7) compared to the scenario under MCAR (row 1), providing that the missing rate for genotype "11" is preferentially higher or lower.

The remaining missing patterns (row 8–13) are when all family members are missing informatively. By excluding incomplete triads from the analysis, the TDT and HRR are no longer valid for testing linkage and association. However, incorporation of dyads and monads reduced such biases. We also found that power of the TDT and HRR excluding incomplete triads can be either reduced (row 8–10) or inflated (row 11–13) compared to the scenario under MCAR (row 1) when the missing rate for genotype 11 is preferentially higher or lower.

## Discussion

The TDT was introduced to test for linkage and association between a marker and a putative disease locus using case-parent triads. Assuming that offspring genotypes are missing complete at random, the TDT excluding incomplete triads is considered a valid test even when parental genotypes are missing informatively. However, if a specific genotype is missing preferentially for parents, it is also likely to occur for the affected offspring.

In this article, the conditional distribution of ascertained triads allowing informative missingness for offspring genotypes as well as their parental genotypes was derived. Through mathematical calculations, we prove that the TDT and HRR do not provide a valid test for linkage and association under such a missing pattern. In addition, we confirmed our conclusion based on computer simulations, since we observed inflated type I error and/or reduced power for the TDT and HRR under such scenarios. Therefore, if the missing pattern for offspring genotypes is not confirmed to be completely at random, a significant result from the TDT or HRR using only complete triads does not assure true association between the marker and a putative disease locus.

## Competing interests

The author(s) declare that they have no competing interests.
